# Developmental Role of Zebrafish Protease-Activated Receptor 1 (PAR1) in the Cardio-Vascular System

**DOI:** 10.1371/journal.pone.0042131

**Published:** 2012-07-30

**Authors:** Elin Ellertsdottir, Peter R. Berthold, Mohamed Bouzaffour, Pascale Dufourcq, Vincent Trayer, Carole Gauron, Sophie Vriz, Markus Affolter, Christine Rampon

**Affiliations:** 1 Biozentrum der Universität Basel, Growth and Development, Basel, Switzerland; 2 Univ Paris Diderot, Sorbonne Paris Cité, Paris, France; 3 Center for Interdisciplinary Research in Biology, College de France, Paris, France; 4 Centre national de la recherche scientifique UMR 7241 and Institut National de la Santé et de la Recherche Médicale, Paris, France; Deakin School of Medicine, Australia

## Abstract

Thrombin receptor, F2R or PAR1 is a G-protein coupled receptor, located in the membrane of endothelial cells. It has been initially found to transduce signals in hemostasis, but recently also known to act in cancer and in vascular development. Mouse embryos lacking PAR1 function die from hemorrhages with varying frequency at midgestation. We have performed a survey of potential PAR1 homologs in the zebrafish genome and identified a teleost ortholog of mammalian PAR1. Knockdown of *par1* function in zebrafish embryos demonstrates a requirement for Par1 in cardio-vascular development. Furthermore, we show that function of Par1 requires the presence of a phylogenetically conserved proteolytic cleavage site and a second intracellular domain. Altogether our results demonstrate a high degree of conservation of PAR1 proteins in the vertebrate lineage in respect to amino acid sequence as well as protein function.

## Introduction

The protease-activated receptors (PARs) form a class of G-protein-coupled receptors (GPCRs) activated by a unique proteolytic cleavage in the extracellular N-terminus. The PAR family member PAR1, also termed f2r for coagulation factor II receptor, is constitutively expressed at the surface of endothelial cells. It has been shown to be cleaved by a highly selective group of serine proteases that includes thrombin, plasmin [1], coagulation Factor Xa [2] and activated protein C [3]. Recent studies have shown that platelet matrix metalloprotease-1 (MMP-1) also cleaves PAR1, but at a different site [4], [5]. Cleavage unmasks a short peptide sequence that interacts with the second extracellular loop, acting as a tethered ligand for the receptor [6]. The proteolytic activation of PAR1 induces transmembrane signalling to internally located G proteins [7]. MMP-1 creates a longer tethered ligand, which activates a distinct spectrum of G protein pathways [5]. Targeted disruption of *Par1* in mice resulted in bleeding at multiple sites, cardiovascular failure and partial embryonic lethality, likely involving endothelial defects that did not impair hemostasis [8], [9]. Whereas vasculogenesis (i.e. the formation of the primitive vascular plexus from endothelial progenitors) was normal, *Par1* mutants displayed abnormal yolk sac vascular pattern due to disorganization or delayed remodelling of the primitive vascular network, as well as some breaks in the sinus venous wall. Surprisingly, half of the *Par1* ^−/−^ embryos survived and became grossly normal adult mice with no bleeding diathesis. Reconstitution of a PAR1 transgene driven by an endothelial-specific promoter rescued the *Par1* ^−/−^ embryonic lethality [9]. Numerous *in vitro* data showed that PAR1 contributes to many functions, such as alteration of vascular tone and permeability, angiogenesis, smooth muscle cell proliferation, production of extracellular matrix, and alteration of gene expression including that for adhesion molecules, chemokines, cytokines and recently the EHT (endothelial-to-hematopoietic transition) (for review see [10], [11], [12]. The irreversible N-terminus cleavage and activation of PAR1 necessitates rapid desensitization by phosphorylation, internalization and receptor level recovery from an internal pool of PAR. *De novo* synthesis is thereafter initiated to restore normal internal level [13], [14].

The zebrafish, due to the facility of observing and manipulating early embryos, is an excellent model for investigating the molecular basis of vertebrate development, including vascular morphogenesis *in vivo* [15]. The zebrafish vascular system develops in a similar fashion to other vertebrates and because of its small size, the zebrafish embryo can absorb enough oxygen through passive diffusion from water to survive for several days even in the complete absence of a functional vasculature.

In this study, we have examined the possible involvement of Par1 in vascular morphogenesis. We reported the characterization of a zebrafish Par1 protein orthologues to those previously identified in other species. Moreover, zebrafish *par1* knockdown led to heart dysfunction and vascular defects. We also demonstrated that the N-terminus and the secondary loop domains of Par1 are necessary for normal cardiovascular development clarifying the role of Par1 during vascular embryonic development.

## Results and Discussion

### PAR1 is highly conserved throughout evolution

To identify PAR1 homologues in the zebrafish, we performed a BLAST search for a zebrafish homologue of the human PAR1 receptor in the most current zebrafish genome database (Ensembl, Zv9). Similar to previously published [16], we identified a zebrafish homologue, Par1 (ENSDARG00000060012, A2BIP6, BX950872.7). Zebrafish Par1 displays 61.6% consensus and 41.4% identity to human PAR1. As described for other species, zebrafish Par1 hydropathy profile analysis revealed a putative seven transmembrane structure (**Fig. S1A–C**). Looking for characteristic features of PARs in zebrafish Par1, we identified the sequence R^28^/SFSGFF, which is very similar to the R^41^/SFLLRN motif at which human PAR1 is cleaved and activated by thrombin [17]. Whereas the N- and C- terminus are moderately similar, the intra- and extra-cellular loops are highly conserved. In particular, the second extracellular loop (the ligand activation site in other species) and the second intracellular loop (important for signal transduction) are strikingly similar (**Fig. S1C**). Phylogenetic analysis also showed that zf-Par1 is most closely related to PAR1 of other teleostei and *Xenopus* (**Fig. S1B**). Our analysis is consistent with the recently published study from Xu et al [16]. The expression pattern during early zebrafish development reported by this group suggests a role of Par1 in haematopoiesis and vasculogenesis. They described *par1* expression in the intermediate cell mass and later in the posterior blood islands (PBI), the heart and veins. Xu et al also found specific expression from 2 days postfertilization (dpf) onward in various organs including the pancreas, the pronephric duct and the gut [16]. Their expression data also indicate maternal contribution of the mRNA, resembling that of the mammalian *PAR1* gene.

Additionally to this zebrafish *par1* gene, several potential duplicate genes appeared during the search. This issue had previously been addressed, asserting high occurrence of gene duplication for all the PAR family members [18]. A later revision clarified these data as discrepancy between the database versions [16]. We found possible uncharacterized proteins resembling Par1. We performed a phylogenic and extended-homology alignment of the potential duplicate genes versus the zebrafish Par1 (F2r), Par2a (F2rl1.1), Par2b (F2rl1.2) and Par3 (F2rl2) receptors (**Fig. S2**).

The initially identified zebrafish Par1 protein is more closely related to Par1 orthologous in both fish and mammals, however the specific relationship of the potential paralogues to zf Par1 was highly dependent on method of analysis, but always scored higher conservation to Par1 than Par2a/b (**Fig. S2A**). It is noteworthy that in several simulations, the Par1 paralogues were often grouped with one of the Par1 paralogues in both *Takifugu rubripes* and *Tetraodon nigroviridis*. Moreover, highly conserved sequences were found in putative transmembrane domains of the Par1 protein. There is a clear homology between Par1 and Par2a/b, while for the paralogue candidates; N-terminal and C-terminal are different from these three proteins (**Fig. S2B**). Furthermore, human *PAR1* and *PAR2* genes are located on the same chromosome (5q13), whereas the here addressed zebrafish *par1* and *par2(a/b)* genes, reside on different chromosomes (5 and 21, respectively). The candidate duplicate genes are located in the same 0.2 Mb region as *par2a* and *par2b* (**Fig. S2B**), further suggesting that they are either coding for Par2-like proteins or that *par1* was translocated after duplication. The mentioned putative paralogues needs to be further characterized.

Interestingly, expression analysis of other members of the PAR family in the zebrafish do not indicate that there could be a redundant functional gene of the same family in endothelial cells [16]. An *in situ* hybridization study demonstrated that zebrafish *par1* and *par2a-b* genes display distinct spatial and temporal expression patterns during development. In addition, *par3* was not detected before 3 dpf by RT-PCR [16]. In mammals, four protease-activated receptors constitute the PAR family. In mouse, PAR1 and PAR4 receptors serve at least partially redundant roles in endothelial cells and together are necessary for the measurable thrombin responses [19]. Like others, we failed to identify a zebrafish homolog of the human or mouse PAR4 gene.

### Genetic knockdown of *par1* disturbs cardio-vascular maturation

**Figure 1:**
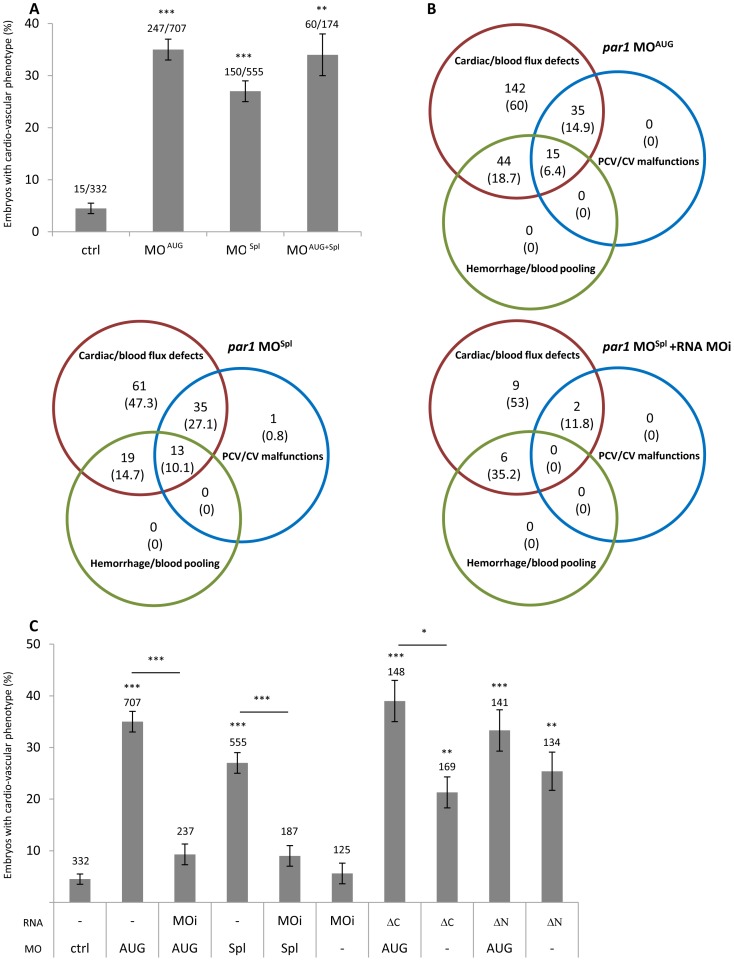
*par1* knockdown induces cardio-vascular phenotype at 48 hpf. (**A**) cardio-vascular phenotype in control and *par1* morphants. n values were indicated on the top of each bar of the graph. (**B**) Venn diagram representation of three classes of vascular failure. n values (% of embryos/embryos with cardio-vascular phenotype) were indicated. (**C**) cardio-vascular phenotype of morpholino and/or RNA injected embryos. Three *par1* mRNA mutants were used. n values were added on the top of each bar of the graph. Average ± error bars from ≥3 independent experiments are presented. * p<0.05; ** p<0.01; *** p<0.001. ctrl, control; MOAUG, *par1* MO^AUG^; MOSpl, *par1* MOSpl; MOi, *par1* mRNA morpholino-insensitive; ΔC, *par1* mRNA morpholino-insensitive lacking intracellular domain; ΔN, *par1* mRNA N terminus deleted.

The evolutionary conserved expression of *par1* mRNA suggests fundamental functions for this gene. To investigate the function of Par1 in zebrafish during early embryonic development, we used morpholino knockdown as a reverse genetic approach. Our morpholinos target the region surrounding the translation start codon of transcripts (*par1* MO^AUG^) or inhibit the splicing of pre-mRNA between the exon 1 and intron 1 (*par1* MO^Spl^). RT-QPCR, performed on morphants embryos injected with the splice-targeting morpholino, revealed a threefold increase of the non-spliced form. At 48 hours post fertilization (hpf), 35% (MO^AUG^) and 27% (MO^Spl^) of *par1* morphants displayed a cardio-vascular phenotype (**Fig. 1A**). Our phenotypic analysis of *par1* morphants in zebrafish matched well with the partial mouse embryonic phenotype and lethality previously described by others [8], [9]. We observed that *par1* morphants with a cardio-vascular phenotype, had weak heart rates associated with slowed to no blood flow. Comparison of *par1* morphants showing this phenotype revealed two additional classes of vascular failures (**Fig. 1B**); haemorrhage/blood pooling and PCV/CV malfunctions. We never observed a higher percentage of phenotypes by injecting higher concentration of the morpholino (than 4 ng) or by co-injection of the two morpholinos. Lower concentration of the morpholino revealed concentration-dependent numbers of embryos with a phenotype (**Table 1**). Co-injections with Rhodamin Dextran indicated no phenotypic variation due to variations in injection volume (data not shown).

**Table 1:** Dose dependence of *par1* MO^AUG^ phenotype at 2 dpf.

	Dose (ng)	Embryos (n)	Embryos with phenotype/n (%)
Ctrl MO	4	332	4,5±1
*par1* MO^AUG^	1,3	135	12,2±2,8
	2	106	17,2±4,1
	4	332	35,0±2,0

### Intact cleavage site and second intracellular domain are required to rescue *par1* knockdown

In order to test the specificity of our antisense strategy, we designed a morpholino-insensitive *par1* mRNA construction which is mutated at the MO target, named *par1*-MOi (**Fig. S1D**). We observed that co-injection of *par1*-MOi mRNA with *par1*-MO^AUG^ or *par1*-MO^Spl^ was able to rescue the cardio-vascular phenotype, allowing the embryos to develop normally. This provides strong evidence that our observations are specific to *par1* knockdown (**Fig. 1B–C** **and** **3A**). Involvement of zebrafish Par1-conserved domains in the cardio-vascular phenotype was evaluated with two mRNA constructs: *par1*-ΔN, where the N-terminus cleavage site is deleted, and *par1*-ΔC, lacking the second intracellular domain required for signalling (**Fig. S1D**). The former cannot be activated by classical cleavage, whereas the latter should have lost the ability to transduce any signal. Co-injection of *par1*-ΔC or *par1*-ΔN mRNA with *par1*-MO^AUG^ was unable to rescue the vascular phenotype, respectively (**Fig. 1C**). These results indicate that the Par1 protein acts during vascular development in the zebrafish embryo and that this activity requires proteolytic cleavage and the intracellular domain as in mammals. Furthermore, overexpression of Par1-ΔC or -ΔN in wild type embryos suggests a dominant negative effect of these truncated receptors.

### Heart beat weaker and blood flow slower in *par1* knockdown

**Figure 2:**
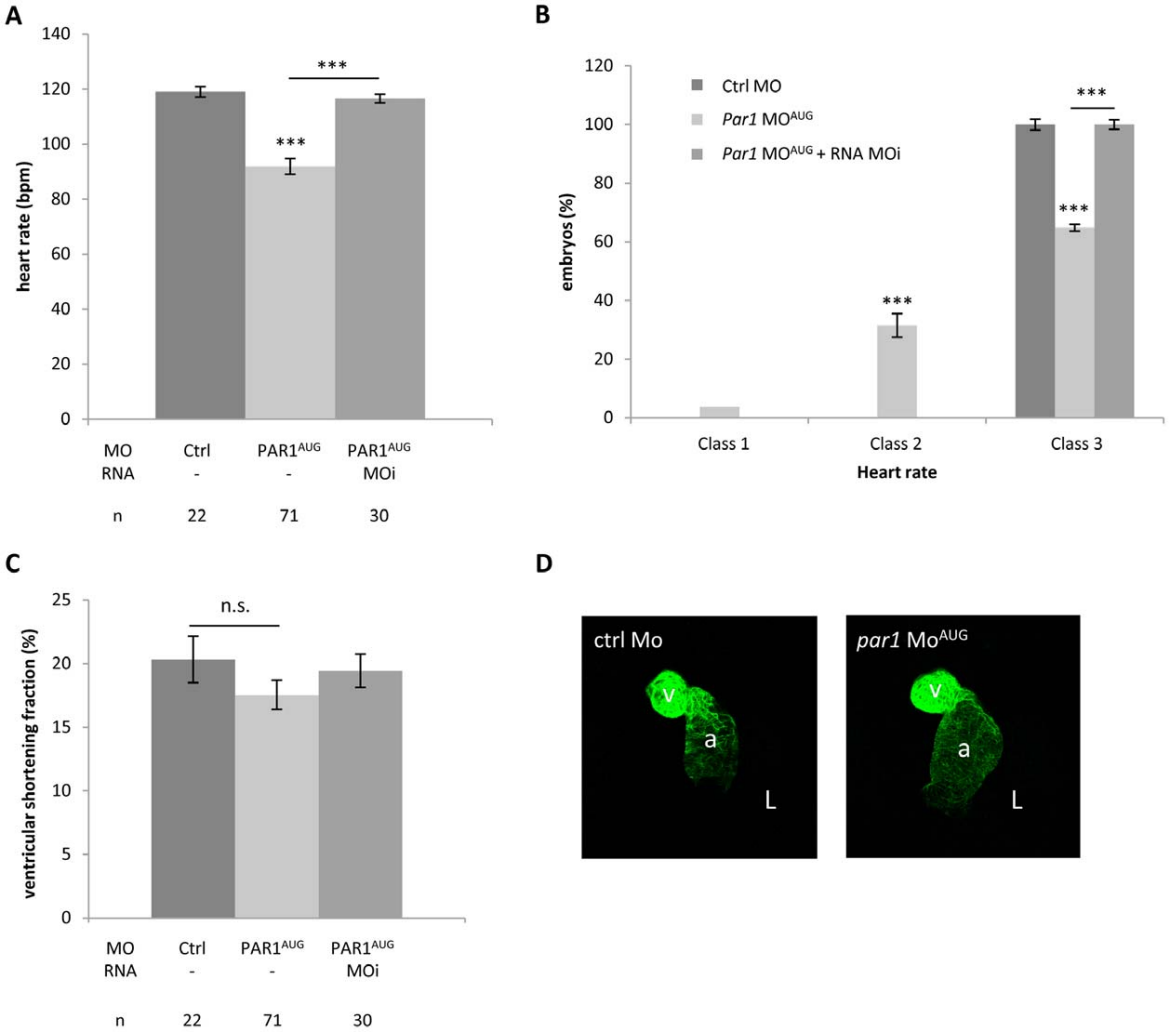
Slow heart rate in *par1* morphants. (**A**) Heart rates were determined at 48 hpf and significance was described from an ANOVA-test statistic. The heart rate of *par1* morphants was significantly lower than controls. *** p<0.001. n values were indicated. (**B**) Classification of heart rate. Class 1: heart shows no heart rate; class 2: heart presents lower heart rate <90 bpm; class 3: heart rate seems normal >90 bpm. (**C**) Ventricular shortening fraction (**D**) The heart is labeled with anti-myosin heavy chain antibody in 48 hpf embryos. Ventral view with the head on the top. a, atrium; v, ventricle, L; embryo left.

As the heart starts to beat in the one day old zebrafish embryo, no apparent difference was seen before between Ctrl MO and *par1* MO^AUG^ injected embryos. At 48 hpf heart rate counts were determined and *par1* MO^AUG^ morphants (injected with a dose of 4 ng) displayed significantly lower heart rate compared to controls (**Fig. 2A**). This difference is maintained at 3 dpf. We classified the heart beat rate into three classes; class 1: absent; class 2: heart rate<90 beats per minute (bpm) and class 3: with normal heart rate>90 bpm. Interestingly, injection of *par1* MO^AUG^ led to class 1 (3.7%±2) and 2 (31.5%±6 with a mean at 72.9±4.7 bpm) (**Fig. 2B**). Classes 1 and 2 were never observed in Ctrl morphants. Furthermore, *par1* morphants heart rate in the normal class where significantly lower (105.4±1.3 bpm) than in controls (117.9±2.8 bpm) (p<0.001). We observed that co-injection of *par1*-MOi mRNA with *par1*-MO^AUG^ was able to rescue this heart beat phenotype (**Fig. 2A–B**). Moreover, in embryos with lower cardiac heart rates (<90 bpm), no obvious abnormalities in the heart morphology can be detected before the functional defect appears. We never observed retrograde flow or fibrillation. Ctrl and *par1* morphant heart beats regularly and co-ordinately with a 1∶1 ratio of atrial to ventricular beats. Furthermore, anti-myosin heavy chain immunostaining revealed that cardiac looping was not affected by *par1* MO^AUG^ injection (**Fig. 2D**). The atrium and the ventricle can be clearly distinguished and constriction was evident at the atrioventricular boundary. In the morphant embryo, the atrium was slightly enlarged. We quantified cardiac contractility by measuring the ventricular shortening fraction (VSF) [20]. VSF of the Ctrl morphant and the *par1* MO^AUG^ injected embryo was not significantly different (**Fig. 2C**). Our results demonstrate a requirement for Par1 in cardiac function that is consistent with *in vitro* studies that have linked PAR1 activation to an increased contractile response in adult cardiomyocytes [21], [22].

**Figure 3:**
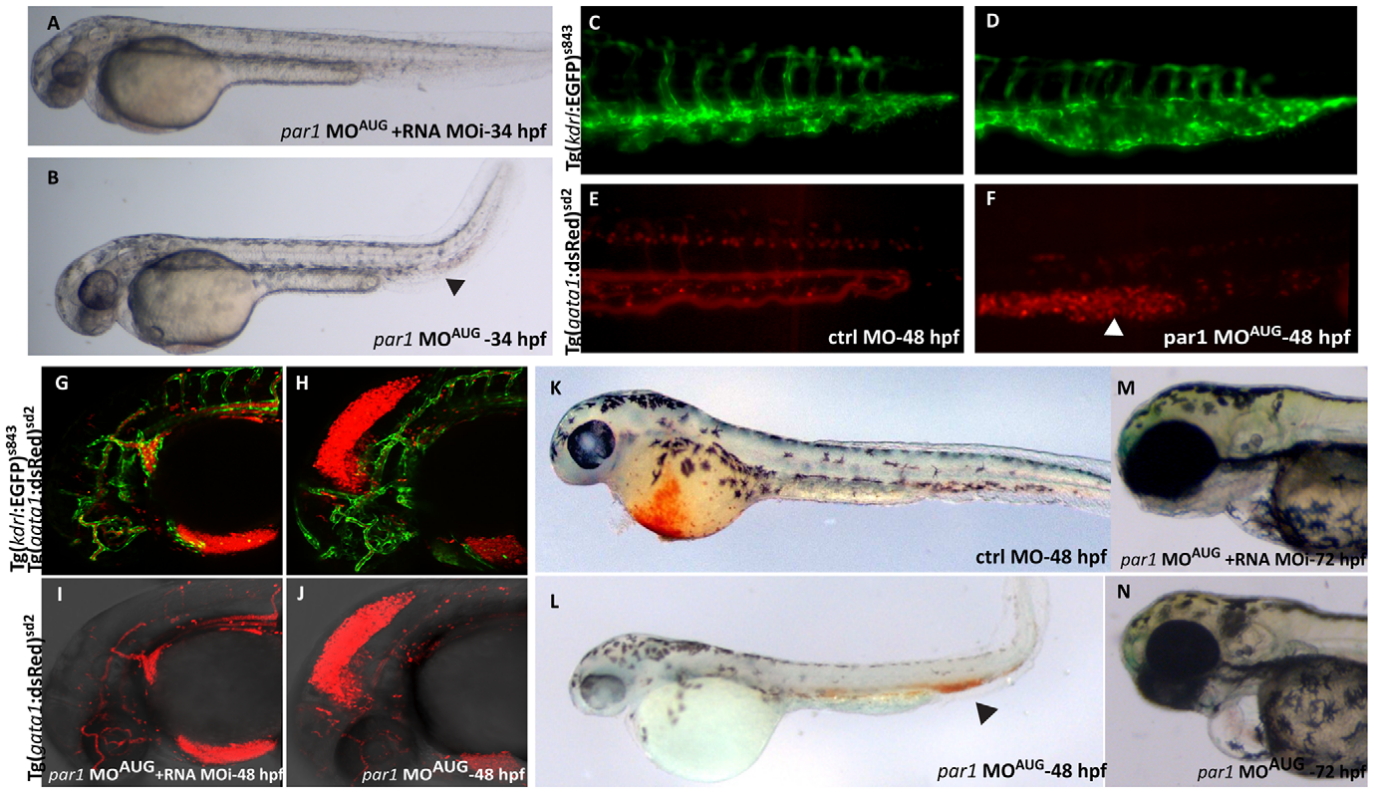
*par1* knockdown causes ICM blood pooling, heart edema and head hemorrhage. (**A**) 34 hpf rescued embryo with heart beat and blood flow, resembling a ctrl MO embryo at this stage. (**B**) *par1* morphant showing blood pooling (arrowhead) with bend tail and heart edema. (**C–F**) The posterior part of the embryo at 48 hpf. The ctrl MO embryo has dorsal aorta, CV, ISVs and arteries with expanded lumen with blood flow as seen in the double transgenic background (**C and E**). The *par1* morphant shows ISVs, arteries, dorsal aorta and CV, but the blood cells are pooled in the CV (arrowhead in **F**) and the vein is bulged and malformed (asterisk in **D**). (**G–J**) Head of 48 hpf double transgenic embryos, displaying endothelial cells in green fluorescence and blood cells in red fluorescence (Tg(*kdrl*:EGFP)^s843^; Tg(*gata1*:dsRed)^sd2^). The blood vessels in the head of both the rescued embryo and the morphant are developed normally, but there is a leakage of blood cells into the mesencephalon of the morphant embryo, causing hemorrhage. Blood flow in the body of both rescued and morphant embryo was normal (not shown). (**K–L**) O-dianisidine staining at 48 hpf. (**K**) embryo treated with Ctrl MO displaying normal blood circulation; (**L**) embryo treated with *par1*-MO^AUG^ showing reduced level of Hb in the Duct of Cuvier and blood pooling in the tail. (**M–N**) Embryos at protruding mouth stage (72 hpf). Contrary to the rescue embryo (**M**), the *par1* morphant has a slight cardiac edema, no mouth protrusion and no visible swim bladder (**N**).

In association with the heart-beat defect, embryos of classes 1 and 2 had a significant slower blood flow, gradually coming to a complete stop in 20% of these embryos. Furthermore, some *par1* morphants with cardio-vascular phenotypes had haemorrhages (**Table 2**) in the head, around the MtA (Metencephalic Artery) and the MCeV (Middle Cerebral Vein) (**Fig. 3J**), blood pooling in the location of the posterior blood island (PBI) (**Fig. 3B and F**) and hemorrhages and oedemas were apparent around the heart itself (**Fig. 3N**). Blood cell pooling can restrain or completely prevent blood flow. *In vivo* live imaging in the knockdown zebrafish embryo substantiate the internal bleeding in the head, due to haemorrhages from the forming CtA (central arteries) posterior to the MtA (metencephalic artery) and the MCeV (middle cerebral vein), see movie S1, or in the forming CVP (choroidal vascular plexus) anterior to the MtA and the MCeV (data not shown), and all small plexus sprouting from the PHBC (primordial hindbrain channel).

**Table 2:** Hemorrhages/blood pooling localisation.

			n (% embryos/heart beat defective embryos)		
	Embryos (n)	Embryos with cardio-vascular phenotype (n)	Head	Heart	Body
*par1* MO^AUG^	707	248	33 (14)	44 (18)	26 (11)
*par1* MO^Spl^	555	150	24 (16)	21 (14)	18 (12)

**Figure 4:**
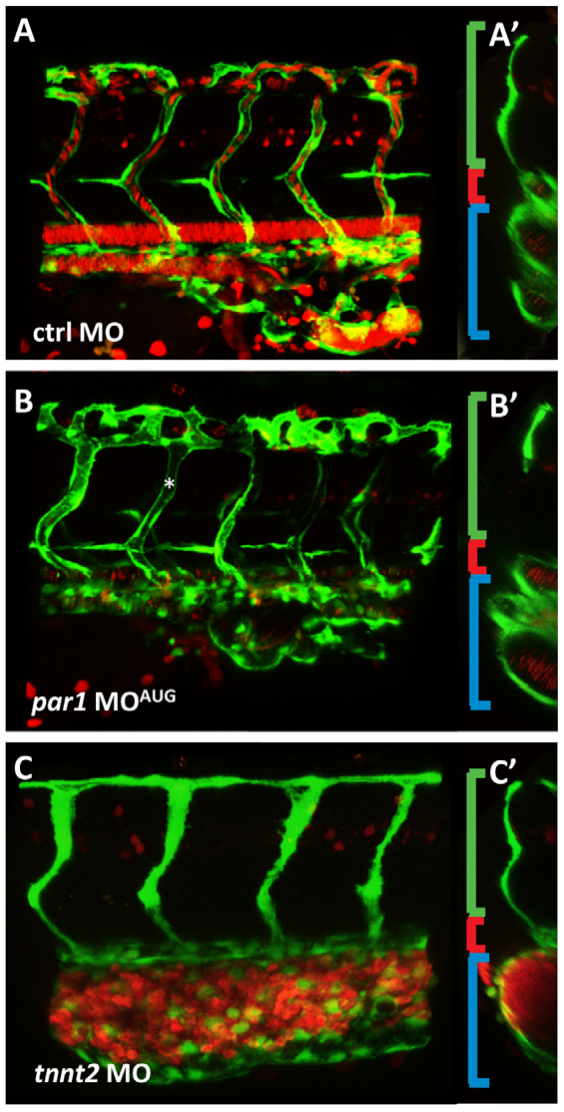
*par1* knockdown impairs vascular remodeling in the cardinal vein. (**A–B**) Lateral views of double transgenic embryos (Tg(kdrl:EGFP)^s843^;Tg(gata1:dsRed)^sd2^) at 35 hpf. (**A**) The intersegmental vessels are connected to the CV and the one intersegmental artery (most posterior) seen in the figure has blood flowing from the dorsal aorta. (**A′**) In the mid-cross section, the intersegmental artery is visible (green bracket), the dorsal aorta can be seen as one tube (red bracket), and the CV is seen as two tubes (blue bracket). (**B**) *par1* morphant lateral view. ISVs are lumenized (asterisk) (**B′**) In cross section, the CV tube does not show a defined number. Due to mislaid vein remodeling the tube is disorganized, cell clusters are apparent instead of a round tube. (**C**) The *tnnt2* knockdown shows no mature inflation of the dorsal aorta and only a minor primary lumen in the ISV. The CV is bulged and full of blood cells in this region. (**C′**) The small dorsal aorta is a clear difference to the *par1* knockdown, additionally to the lack of lumen in the ISVs and blood cells always clogging in the region of origin, PBI.

Interestingly, we observed that the formation of the vascular system was altered in the caudal vascular plexus (**Fig. 3D** **and** **4B**). Caudal vein (CV) cell rearrangement was in some cases not complete (23% of embryos in grades 1 and 2 with MO^AUG^ and 46% with MO^spl^). To further characterize this phenotype, we analysed a double transgenic zebrafish strain expressing EGFP under the promoter of the endothelial specific *kdrl*, and DsRed under the erythroid-specific promoter of *gata1*. While *gata1*-positive cells were located throughout the vascular tree upon injection in control morphants (**Fig. 3C and E**), erythroid cells were enclosed by the vascular tissue in the posterior part of the vein in *par1* morphants with blood pooling in the location of the PBI (arrowhead in **Fig. 3F**). Furthermore, we observed that the CV in this region was dilated and failed to form, as seen with the green fluorescence in Tg(*kdrl*:EGFP)^s843^ embryos (asterisk in **Fig. 3D**). Blood pooling in the posterior body region of the morphant embryo was confirmed by haemoglobin (Hb) localization using O-dianisidine staining (**Fig. 3K–L**). In control morphants, Hb staining was high in the Duct of Cuvier and weak in the tail (**Fig. 3K**). In *par1* morphants with blood clots at PBI, the Hb staining showed that erythrocytes were confined to this region (arrowhead in **Fig. 3L**). Bulged similar dilated posterior caudal plexus has already been described in a different study with more or less severe abnormal blood circulation [23], [24]. In virtual cross sections of confocal scans within the posterior cardinal vein double transgenic embryos (Tg(kdrl:EGFP)^s843^;Tg(gata1:dsRed)^sd2^) the difference between the morphant and the control embryo became clearer. In control embryos the ISV's are inflated at this stage development (35 hpf), the dorsal aorta can be seen as a single tube and the posterior cardinal vein appears as 2 to 3 tubes in the cross section. In *par1* morphants, ISVs are also luminized and the dorsal aorta can be seen as one tube but CV tube does not show a defined number and is somewhat dilated. In comparison in the *tnnt2* morphant which has a complete silent heart phenotype [25], the dorsal aorta was small and the intersegmental vessels (ISVs) were collapsed. The caudal vein in the *tnnt2* morphant is seen as one big tube filled with all the blood cells that migrate into the vessel [26], [27], and clog, due to lack of blood flow. Furthermore, oedemas in the heart of the *par1* morphant were not as severe as in the *tnnt2* morphant (**Fig. 4**). These results suggest a direct requirement for Par1 during posterior cardinal vein remodelling or an indirect function via a slower heart beat rate and a slower blood flow.

Finally, considering that PAR1 modulates endothelial barrier integrity through actomyosin contraction and adherens junction modifications [28], we investigated the effect of Par1 knockdown on VE-cadherin-based adherens junctions using antibodies against VE-cadherin. Staining patterns were similar in the *par1* morphant and in the control morphant (**Fig. S3**), This would indicate intact adherens junctions in the endothelial cells of the *par1* morphant. Yue et al recently discovered that Par1 is a negative regulator of the hematopoietic development. Zebrafish embryos in their knockdown experiments had increased number of hematopoietic cells and in mESC (mouse embryonic stem cells), both primitive and definitive erythroid number a.m., was increased if the F2 receptor was chemically or genetically blocked [12]. One may speculate that the increasing number of erythrocytes causes strain on the heart and the blood vessel, resulting in heart failure and bleeding. According to Yue et al, *in vitro* blocking Par1 causes more endothelial cells to undergo endothelial-hematopoetic-transition and abrogation of junctions in the cells. Our analysis of junctional integrity in the endothelial cells, using the VE-cadherin antibody, revealed no difference between *par1* MO injected embryos and control embryos.

## Conclusion

Our sequence analysis and functional data on Par1 in the zebrafish confirm the protein to be highly conserved in the animal kingdom [8], [9]. The PAR1 receptor is clearly involved in cardiovascular development and vessel maturation. Non-functional PAR1 causes defects in vascular maturation and internal bleeding, as shown with live imaging in the zebrafish knockdown embryo. Finally, we demonstrated that the N-terminus and the secondary loop domains of Par1 are necessary for normal cardiovascular development and that PAR1 function and probably activation and signaling is conserved across vertebrates.

## Materials and Methods

### Ethics and fish care

All protocols in this study were conducted in strict accordance with the French and Swiss guidelines for the care and use of laboratory animals. No specific ethics approval was required for this project, as all zebrafish (*Danio rerio*) used in this study were between 0 and 3 days old. Zebrafish embryos were killed with a bleach solution (sodium hypochlorite 6.15%) added to the culture system water at 1 part bleach to 5 parts water. Zebrafish embryos remained in this solution for at least 5 minutes to ensure death. Given the age of the embryos, pain perception has not yet developed at these earlier stages so this is not considered as a painful procedure. For *in situ* hybridization or immunostaining, zebrafish embryos were killed and fixed on PFA 4%. Zebrafish were maintained and staged according to Westerfield [29]. Experiments were performed using the standard Ab wild type strain. All the transgenic lines used came from zebrafish. The following zebrafish transgenic lines were used: Tg(*flia*:EGFP)^γ1^ [30], Tg(*kdrl*:EGFP)^s843^ [31], and Tg(*gata1*:dsRed)^sd2^ [32].

### Zebrafish PAR1 gene identification

The human PAR1 amino acid (aa) sequence (P25116 UniProt) was used to perform BLAST searches in both Ensembl and NCBI databases. No human tissue was used for this study. We obtained the human sequence from a genebank and performed sequence analysis *in silico*. *In silico* alignment analysis was done by two different approaches, LALIGN (http://www.ch.embnet.org/software/LALIGN_form.html) and Vector NTI AlignX software, and both were used in the determination of a bona fide zebrafish PAR1 homologous. Multiple alignments were likewise done by AlignX or PRALINE [33]. Proteomic analysis and predictions were made with TMHMM, TMPred and Predictprotein.org (ExPASy portal: http://www.expasy.ch/). The clone corresponding to the sequence identified in silico (# CO935967) was ordered from ImaGenes (Berlin, Germany) and re-sequenced to confirm the cDNA.

### Microinjection of morpholino antisense oligonucleotides (MOs)

MOs were purchased from Gene Tools (LLC, Philomath, OR, USA). For the experiments 4 ng of each morpholino were used for injection into one- to two-cell stage zebrafish embryos [34]. *Par1*-specific MO^AUG^ (5′-CCGTCACCAACAGAACCCGCAACAT-3′ ) was used to inhibit Par1 protein synthesis (translational initiation codon is underlined). We also performed some experiments with a splice site directed morpholino MO^spl^ (5′-GAAAGTCTGTAAGCGTCTTACCGTT-3′) that inhibited the splicing of pre-mRNA between the exon 1 and intron 1. (5′-CCGTCAGGAAGACAACGCGCAAGAT-3′) was used as a control MO. For a complete knockdown of the heart beat, an antisense morpholino oligonucleotide targeting the cardiac troponin T translation start codon and flanking 5′ sequence was injected (5′-CATGTTTGCTCTGATCTGACACGCA-3′) [25].

### Quantitative RT-PCR

cDNA synthesis and RT-PCR reaction were performed as previously described [35]. Gene expression level was normalized to *RPL13*. The *Rpl13a* primers were previously described in [36]. Each sample was tested in duplicate. The *par1* primers were *fw* 5′-TGTTACAATGATTAAATGCTGCAA-3′, *rv* 5′-CATCAGTGACGGTGAGGAAA-3′.

### Heart rate counts and ventricular contractibility analysis

Heart rates (beats per minute) were counted manually under Leica stereo microscope M80 for control MO and *par1* MO^AUG^ injected zebrafish embryos at 48 hpf. Embryos were anesthetized 10 sec in tricaine, transferred to a recording chamber, mounted and perfused with embryo medium. The ventricular shorthening fraction (VSF) was measured according to Wang et al [20]. The heart rate (beats/min) and the VSF were recorded by the same operator after a recovery in embryo medium of at least 10 min.

### Hemoglobin staining

*In situ* Hb staining was carried out using O-dianisidine as described by Kwan et al [37]. The presence of yellow-brown coloration indicates the presence of hemoglobin in zebrafish embryos.

### Constructions and mRNA injection

cDNA from clone # CO935967 was cloned in pCS2+ vector. Directed mutagenesis allowed us to construct a cDNA mutated at the MO target (MOi clone for MO insensitive RNA) as well as two deletion mutants ΔC (lacking the 69 C-terminal aa) and ΔN (lacking the first 79 aa). A detailed description of the plasmids is available upon request. mRNA synthesis was performed using the mMESSAGE mMACHINE high yield capped RNA Transcription Kit (Ambion Inc., Austin, TX, USA). 150 ng/ml of transcribed mRNA was injected into one-cell stage embryos. For the rescue experiments, mRNA and morpholino were co-injected.

### Immunofluorescence

Zebrafish anti-Ve-cadherin antibody was used diluted 1∶500 [38]. Alexa 688 goat anti-rabbit IgG were diluted 1∶1000. Anti-myosin heavy chain antibody (Millipore, Temecula, CA, USA) was used diluted 1∶200 to visualize the heart at 48 hpf.

### Microscope imaging

Living zebrafish embryos were mounted in methyl-cellulose on a slide (with slope) and imaged with a LeicaDFC 420C camera on a Leica MZF III binocular. Photos were processed with Adobe Photoshop CS4.

### Confocal imaging

Living zebrafish embryos were mounted in 0.8% low melting agarose in a small Petri dish with cover slip bottom, covered with egg water, and imaged on the Leica TCS SP5 confocal microscope. Objective ×20 with 2 times zoom or ×63 were used. Fixed embryos were mounted in fluorescent mounting medium and imaged on a slide with a cover slip on the same Leica SP5. All images were analysed with Imaris 7.1.1 (Bitplane) Software. All images are compressed z-stalk of an embryo. For overnight imaging, embryos were mounted as described above and kept in a chamber (The Box) with constant temperature of 28°C.

### Statistical analysis

For Fig. 1, all values are expressed as means ± standard error. Error bars are standard errors estimated as √(*p*(1−*p*)/*n*) where *p* is the proportion of embryos exhibiting a phenotype and *n* the total number of embryos investigated (or 1/*n* when *p* = 0 or 1). Comparisons were performed by chi-squared test. For Fig. 2, comparisons between multiple groups were performed by 1-way ANOVA followed by Tukey's tests. Significance level was set at P<0.05.

## Supporting Information

Figure S1 **Par1 is highly conserved throughout evolution.** **(A**) Alignment of PAR1 protein sequences; black background: identical amino acid; grey background: conservative change; transmembrane segments and functional domains are indicated. (**B**) phylogenetic tree (**C**) Secondary structure prediction for zebrafish Par1 conforms to a seven-pass membrane receptor structure. Alignment and comparison between functional domains of human PAR1 and zebrafish Par1; black box: thrombin cleavage site; green letters: tethered ligand domain; underlined: hirudin–like sequence; TM1: transmembrane domain 1. (**D**) Three *par1* mRNA mutants were designed: *par1*-MOi, morpholino-insensitive; *par1*-ΔN N-terminus deleted; and *par1*-ΔC, a *par1*-MOi lacking the intracellular domain. Asterisks indicate the morpholino-insensitive sequence on *par1* mRNA.
(TIF)

Figure S2 **Par1 putative paralogues.** (A) Ensembl Phylogenetic tree. (B) Location and sequence analysis (C) Hierarchical multiple sequence alignment (PRALINE) of zebrafish PARs and putative paralogues.
(TIF)

Figure S3 **The endothelial adherens junctions appear intact in** ***par1*** **knockdown.** (**A–C**) Lateral views after labelling of adherens junctions in the ISVs with anti ve-cadherin antibody (green) in a (Tg(*kdrl*:EGFP)^s843^) (red) transgenic embryo. (**D–F**) Immunolocalization of ve-cadherin alone. (**E–F**) Ve-cadherin labelled junctions appeared normal at 33 hpf in *par1* morphants.
(TIF)

Movie S1 **Hemorrhage in the head of a** ***par1*** **morphant.** Live imaging of a rescued double transgenic embryo (Tg(*kdrl*:EGFP)^s843^;Tg(*gata1*:dsRed)^sd2^, injected with MO*^par1^* and RNA MOi) to the left, and *par1* morphant to the right. Live imaging on a SP5 Leica confocal, overnight imaging from 34 hpf to 46 hpf, z-stalks taken every 20 minutes, objective ×10, zoom ×2. Notice how fast the blood cells leak out of the CtA (central artery) in the morphant.
(AVI)
