# Supplementary figures and images for: Developmental Role of Zebrafish Protease-Activated Receptor 1 (PAR1) in the Cardio-Vascular System
Source: PLoS One. 2012 Jul 30;7(7):e42131. doi: 10.1371/journal.pone.0042131 (PMC3408399; doi:10.1371/journal.pone.0042131)

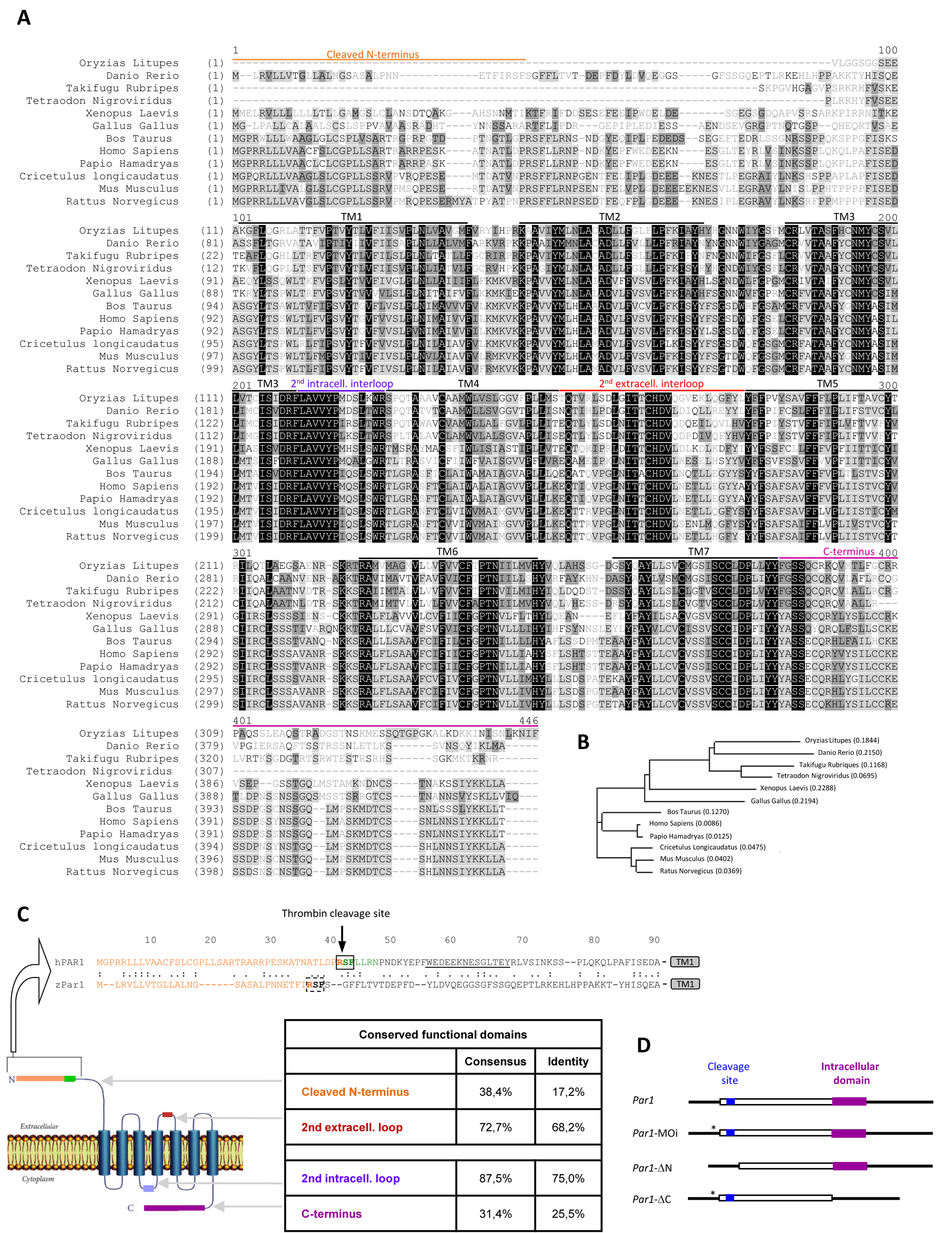

Supplement: Figure S1 — Par1 is highly conserved throughout evolution. (A) Alignment of PAR1 protein sequences; black background: identical amino acid; grey background: conservative change; transmembrane segments and functional domains are indicated. (B) phylogenetic tree (C) Secondary structure prediction for zebrafish Par1 conforms to a seven-pass membrane receptor structure. Alignment and comparison between functional domains of human PAR1 and zebrafish Par1; black box: thrombin cleavage site; green letters: tethered ligand domain; underlined: hirudin–like sequence; TM1: transmembrane domain 1. (D) Three par1 mRNA mutants were designed: par1-MOi, morpholino-insensitive; par1-ΔN N-terminus deleted; and par1-ΔC, a par1-MOi lacking the intracellular domain. Asterisks indicate the morpholino-insensitive sequence on par1 mRNA. (TIF) [file pone.0042131.s001.tif]

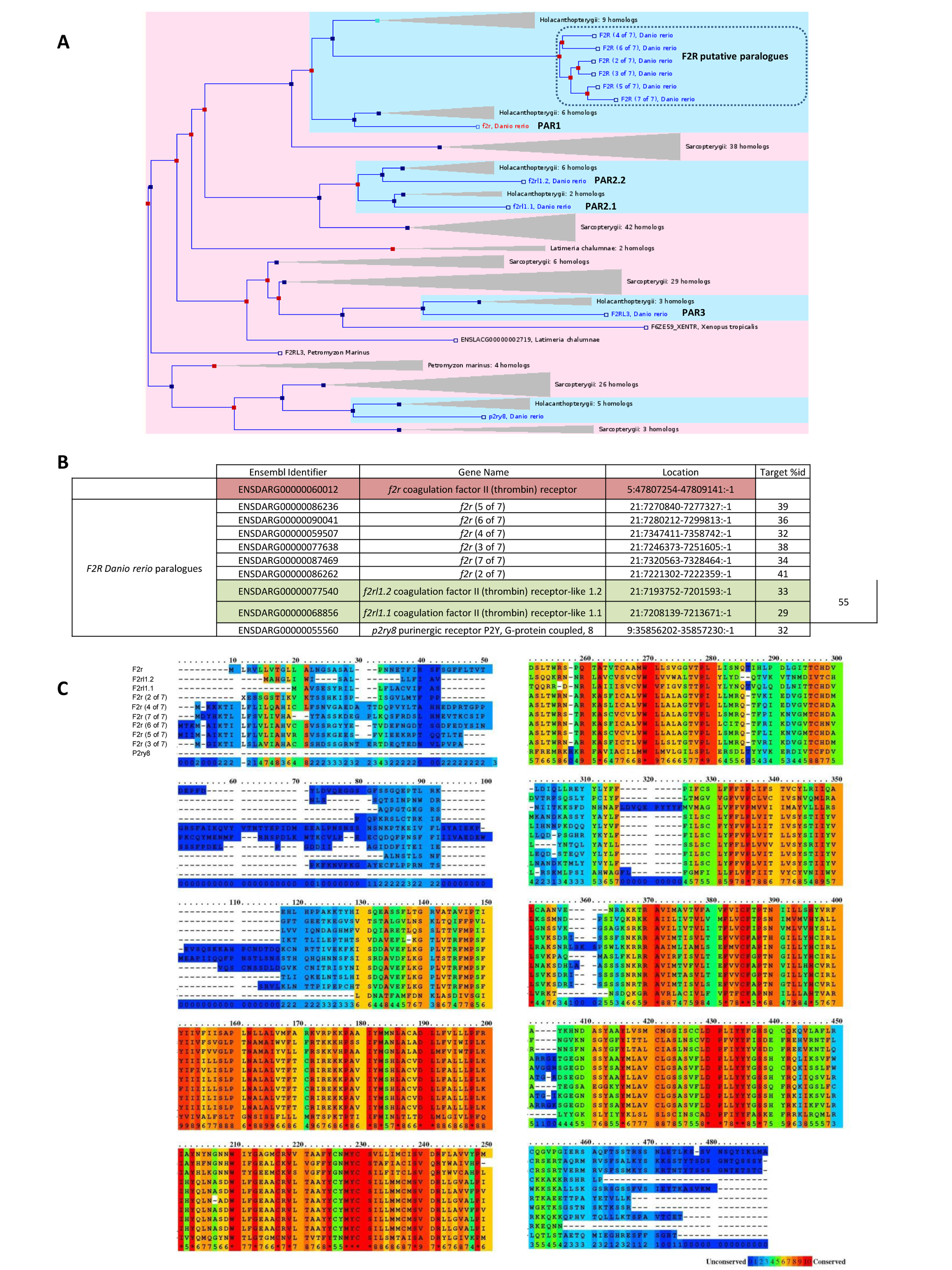

Supplement: Figure S2 — Par1 putative paralogues. (A) Ensembl Phylogenetic tree. (B) Location and sequence analysis (C) Hierarchical multiple sequence alignment (PRALINE) of zebrafish PARs and putative paralogues. (TIF) [file pone.0042131.s002.tif]

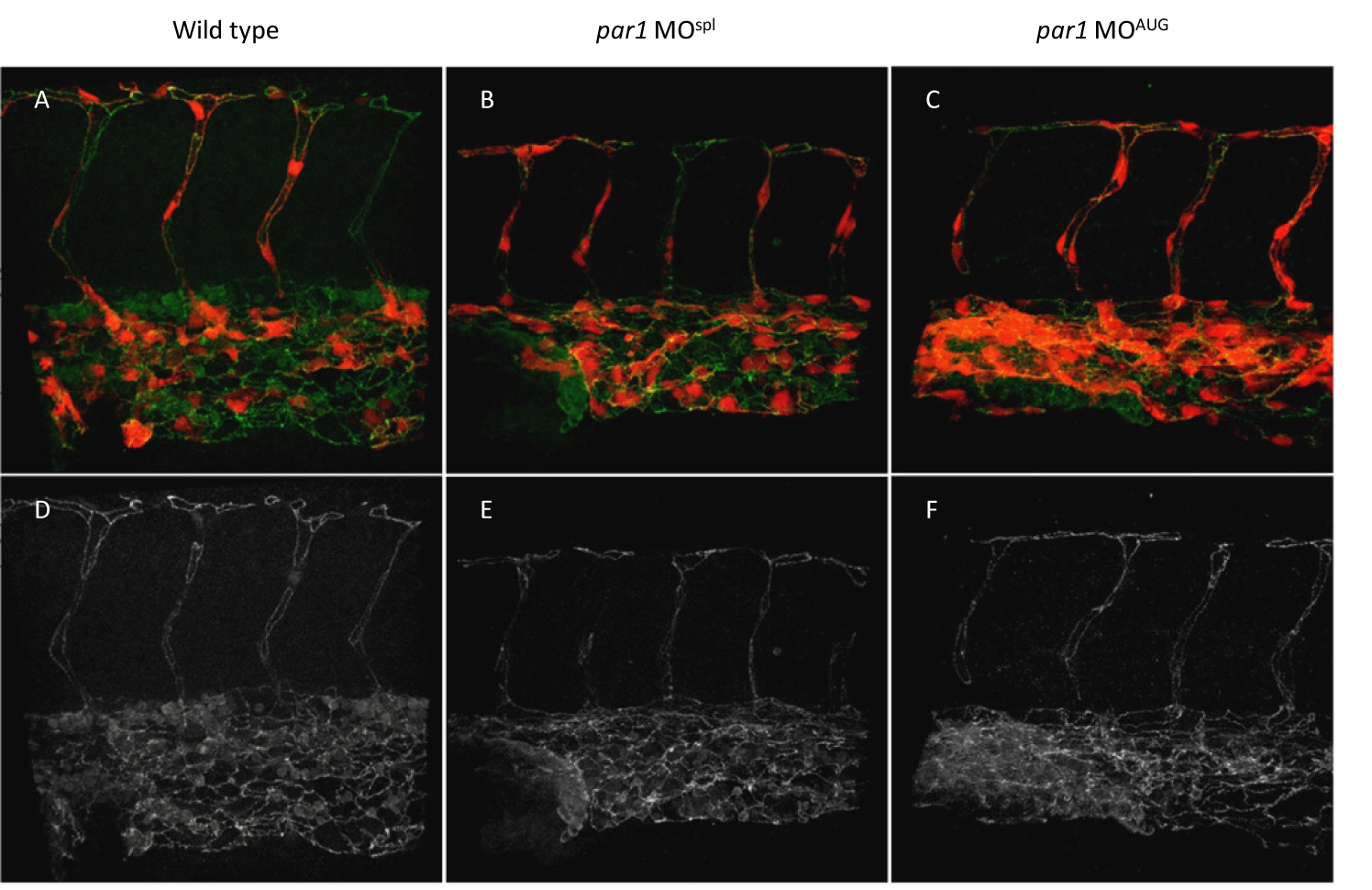

Supplement: Figure S3 — The endothelial adherens junctions appear intact in par1 knockdown. (A–C) Lateral views after labelling of adherens junctions in the ISVs with anti ve-cadherin antibody (green) in a (Tg(kdrl:EGFP)s843) (red) transgenic embryo. (D–F) Immunolocalization of ve-cadherin alone. (E–F) Ve-cadherin labelled junctions appeared normal at 33 hpf in par1 morphants. (TIF) [file pone.0042131.s003.tif]
